# Greater Endothelial Apoptosis and Oxidative Stress in Patients with Peripheral Artery Disease

**DOI:** 10.1155/2014/160534

**Published:** 2014-05-20

**Authors:** Andrew W. Gardner, Donald E. Parker, Polly S. Montgomery, Danuta Sosnowska, Ana I. Casanegra, Zoltan Ungvari, Anna Csiszar, William E. Sonntag

**Affiliations:** ^1^Reynolds Oklahoma Center on Aging, Donald W. Reynolds Department of Geriatric Medicine, University of Oklahoma Health Sciences Center (OUHSC), Oklahoma City, OK 73117, USA; ^2^Veterans Affairs Medical Center, Oklahoma City, OK 73104, USA; ^3^Department of Biostatistics and Epidemiology, OUHSC, Oklahoma City, OK 73117, USA; ^4^Cardiovascular Section, Department of Medicine, OUHSC, Oklahoma City, OK 73117, USA

## Abstract

We compared apoptosis, cellular oxidative stress, and inflammation of cultured endothelial cells treated with sera from 156 subjects with peripheral artery disease (PAD) and 16 healthy control subjects. Furthermore, we compared circulating inflammatory, antioxidant capacity, and vascular biomarkers between the two groups. The PAD group had a 164% higher value for endothelial cell apoptosis (*P* < 0.001) and a 62% higher value for endothelial cellular reactive oxygen species production (*P* < 0.001) than the control group. Furthermore, the PAD group had lower systemic antioxidant capacity measured by hydroxyl radical antioxidant capacity activity (*P* < 0.001), higher inflammatory and vascular measures of high-sensitivity C-reactive protein (*P* < 0.001), interleukin-8 (*P* < 0.001), serum amyloid A (*P* < 0.001), vascular cell adhesion molecule-1 (*P* < 0.001), adiponectin (*P* < 0.001), apolipoprotein B (*P* = 0.013), apolipoprotein CIII (*P* = 0.035), lower vascular endothelial growth factor-A (*P* < 0.001), and hepatocyte growth factor (*P* < 0.001) than the control group. Subjects with PAD have greater endothelial apoptosis and oxidative stress than control subjects with low burden of comorbid conditions and cardiovascular risk factors. Furthermore, subjects with PAD have lower systemic antioxidant capacity and angiogenic measures and higher circulating inflammatory parameters.

## 1. Introduction

Peripheral artery disease (PAD) is prevalent in eight million men and women in the United States [1] and in more than 12% of community dwelling people aged 65 years and older [2]. PAD is associated with increased prevalence of coexisting diseases in the coronary, cerebral, and renal arteries [2, 3]. More than 60% of those with PAD have concomitant cardiovascular and/or cerebrovascular disease [3], thereby contributing to their elevated rates of cardiovascular mortality [4, 5]. The cost associated with PAD is similar to or higher than cardiac dysrhythmias, congestive heart failure, and cerebrovascular disease, averaging $3.9 billion for total Medicare paid PAD-related care annually [6], primarily due to repeat revascularization procedures and recurring hospitalization admissions [7]. Many of those with PAD are physically limited by ambulatory leg pain, resulting in ambulatory dysfunction [8], impaired physical function [9, 10], lower physical activity levels [11, 12], and even worse health-related quality of life scores than in individuals with coronary artery disease and congestive heart failure [13]. Furthermore, patients with PAD have increased rates of functional decline and mobility loss compared to those without PAD [9, 10, 14].

Endothelial dysfunction is an early marker of vascular dysfunction prior to the development of structural changes and clinical symptoms [15–21], contributes to the progression of atherosclerosis [21], and increases the risk of coronary events [15, 17, 21–23]. We [24] and others [21] have found that endothelial function, as measured by the criterion method of brachial artery flow-mediated dilation, is impaired in PAD subjects. Apoptosis, cellular oxidative stress, and inflammation of the endothelium are factors that may contribute to the impaired endothelial function of subjects with PAD. However, the impact of PAD on these endothelial biomarkers compared to healthy subjects is not well understood.

The purpose of this study was to compare apoptosis, cellular oxidative stress, and inflammation of cultured endothelial cells treated with sera from subjects with PAD and healthy control subjects free of atherosclerotic disease with relatively low burden of cardiovascular risk factors. A second aim was to compare circulating inflammatory, antioxidant capacity, and vascular biomarkers between the two groups. We hypothesized that subjects with PAD would have greater endothelial apoptosis, cellular oxidative stress, and inflammation than controls, as well as worse systemic inflammation, antioxidant capacity, and vascular biomarkers.

## 2. Methods

### 2.1. Subjects

#### 2.1.1. Approval and Informed Consent

The procedures used in this study were approved by the institutional review board at the University of Oklahoma Health Sciences Center (HSC) and by the Research and Development Committee at the Oklahoma City VA Medical Center. Written informed consent was obtained from each patient at the beginning of investigation.

#### 2.1.2. Recruitment

Subjects were recruited by referrals from vascular labs and vascular clinics from the University of Oklahoma HSC and the Oklahoma City VA Medical Center for possible enrollment into a randomized controlled exercise rehabilitation study for the treatment of leg pain secondary to PAD [25]. Control subjects were recruited by newspaper advertisements for the assessment of cardiovascular risk factors in individuals without a history of cardiovascular diseases. All subjects lived independently at home.

### 2.2. Medical Screening through History and Physical Examination

Subjects were evaluated at the Clinical Research Center (CRC) at the University of Oklahoma HSC. Subjects arrived at the CRC in the morning fasted but were permitted to take their usual morning medication. Demographic information, height, weight, waist circumference [26], cardiovascular risk factors, comorbid conditions, claudication history, blood and urine samples, and a list of current medications were obtained from a medical history and physical examination at the beginning of the study.

#### 2.2.1. Inclusion and Exclusion Criteria for the PAD Group

Subjects with intermittent claudication were included in this study if they met the following criteria: (a) a history of ambulatory leg pain, (b) ambulation during a graded treadmill test limited by leg pain consistent with intermittent claudication [8], and (c) an ankle-brachial index (ABI) ≤ 0.90 at rest [2] or an ABI ≤ 0.73 after exercise [27]. Subjects were excluded from the PAD group for the following conditions: (a) absence of PAD (ABI > 0.90 at rest and ABI > 0.73 after exercise); (b) noncompressible vessels (ABI > 1.40); (c) asymptomatic PAD determined from the medical screening, ABI test, and graded treadmill test; (d) use of medications indicated for the treatment of claudication (cilostazol or pentoxifylline) initiated within 3 months prior to investigation; (e) exercise tolerance limited by any disease process other than PAD; (f) active cancer; (g) end stage renal disease defined as stage 5 chronic kidney disease; (h) abnormal liver function. A consecutive series of 225 individuals were evaluated for eligibility, and 156 subjects were deemed eligible for inclusion in the PAD group and 69 subjects were ineligible.

#### 2.2.2. Inclusion and Exclusion Criteria for the Control Group

Control subjects were included in this study if they met the following criteria: (a) negative test on the San Diego claudication questionnaire [28], (b) no other ambulatory leg pain, and (c) an ABI between 1.00 and 1.40. Controls were excluded from this study for the following conditions: (a) an ABI < 1.00; (b) noncompressible vessels (ABI > 1.40); (c) any medications, including those for hypertension, dyslipidemia, or diabetes; (d) history of cardiovascular disease, cerebrovascular disease, myocardial infarction, or peripheral revascularization; (e) active cancer; (f) chronic kidney disease; (g) abnormal liver function. Of 23 subjects who were considered for inclusion into the control group, 16 subjects were deemed eligible and 7 subjects were ineligible.

### 2.3. Measurements

#### 2.3.1. Six-Minute Walk Test

Subjects performed an over-the-ground, 6-minute walk test supervised by trained exercise technicians [29]. The pain-free and total distance walked during the test was recorded. The test-retest intraclass reliability coefficient is *R* = 0.75 for distance to onset of claudication pain and *R* = 0.94 for total 6-minute walking distance [29].

#### 2.3.2. Ambulatory Activity Monitoring

Daily ambulatory activity was assessed during seven consecutive days using a step activity monitor (StepWatch3, Ortho Innovations, Inc., Oklahoma City, OK) as previously described [30]. The accuracy of the step activity monitor exceeds 99% ± 1% in patients with claudication [30], and the test-retest intraclass reliability coefficient for the daily ambulatory activity measures ranges from *R* = 0.83 to *R* = 0.94 [30].

#### 2.3.3. Blood Sampling

Venipuncture was done to obtain the blood specimen from an antecubital vein. The blood was collected in vacutainers and then distributed in 0.5 mL aliquots. The serum samples were stored at −80°C and were subsequently batched for analysis.

#### 2.3.4. Endothelial Cell Cultures

We used a cell culture-based bioassay approach utilizing cultured primary human arterial endothelial cells to characterize the endothelial effects of circulating factors present in the sera of subjects. In brief, endothelial cells (purchased from Cell Applications, Inc., San Diego, CA, after passage 4; age of the donors is unknown) were initially cultured in MesoEndo Endothelial Cell Growth Medium (Cell Applications, Inc.) followed by Endothelial Basal Medium supplemented with 10% fetal calf serum until the time of serum treatment, as described [31, 32]. The detector cells used for each in vitro study were from the same donor; thus, interindividual variance is unlikely to contribute to the observed differences. For treatment, fetal calf serum was replaced with serum (10%; for 24–48 h) collected from our subjects, following our published protocols [31]. Cells cultured in Endothelial Basal Medium supplemented with 10% fetal calf serum served as an additional control.

#### 2.3.5. Apoptosis Assay

Cultured endothelial cells were treated with sera from PAD subjects and their respective controls (for 24 h). To determine whether circulating factors present in the sera of PAD subjects exert proapoptotic effects, apoptotic cell death was assessed by measuring caspase activities using Caspase-Glo 3/7 assay kit (Promega, Madison, WI) as previously reported [31].

#### 2.3.6. Cellular Reactive Oxygen Species (ROS) Production

To assess cellular oxidative stress induced by factors present in the sera, hydrogen peroxide (H_2_O_2_) production in detector endothelial cells was measured fluorometrically using the Amplex Red/horseradish peroxidase assay as described [31].

#### 2.3.7. Transient Transfection, Nuclear Factor K-Light-Chain-Enhancer of Activated B Cells (NF-*κ*B) Reporter Gene Assay

To assess cellular proinflammatory effects induced by factors present in the sera, transcriptional activity of NF-*κ*B was tested in serum-treated detector endothelial cells by a reporter gene assay as described [31]. Transfections in endothelial cells were performed using the Amaxa Nucleofector technology (Amaxa, Gaithersburg, MD), as we have previously reported [31].

#### 2.3.8. Serum Antioxidant Capacity

To compare the capacity of antioxidant enzymes and other redox molecules present in the sera of subjects to counterbalance the deleterious effects of oxidative stress, we assessed the Hydroxyl Radical Antioxidant Capacity (HORAC) using the OxiSelect HORAC Activity Assay (Cell Biolabs Inc., San Diego, CA) as previously described [31].

#### 2.3.9. Circulating Inflammatory and Vascular Biomarkers

Circulating cytokines and biomarkers present in the sera was performed using a Milliplex Human Adipokine Magnetic Bead Kit for tumor necrosis factor alpha (TNF *α*), interleukin-1b (IL-1b), interleukin-6 (IL-6), interleukin-8 (IL-8), monocyte chemotactic protein-1 (MCP-1), hepatocyte growth factor (HGF), and nerve growth factor (NGF). A Milliplex Human Cardiovascular Disease (CVD) Panel 1 Kit was used for myeloperoxidase (MPO), matrix metallopeptidase-9 (MMP-9), E selectin, vascular cell adhesion molecule-1 (VCAM-1), intercellular cell adhesion molecule-1 (ICAM-1), and plasminogen activity inhibitor-1 (PAI-1). A Milliplex Human Apolipoprotein Kit was used for apolipoprotein B and apolipoprotein CIII. The Millipore kits were purchased from the EMD Millipore, Billerica, MA. Affymetrix Procarta Immunoassay was used for the detection of serum amyloid A (SAA), vascular endothelial growth factor-A (VEGF-A), and adiponectin. These assays were performed according to the manufacturer's protocols. Sample protein content was determined for normalization purposes by a spectrophotometric quantification method using BCA reagent (Pierce Chemical Co., Rockford, IL).

#### 2.3.10. High-Sensitivity C-Reactive Protein (hsCRP)

A high-sensitivity Near Infrared Particles Immunoassay was used to quantify the concentration of hsCRP from a serum sample of 300 *μ*L. A commercially available device, the SYNCHRON LX-20 (Beckman-Coulter; California, USA), was used to automatically perform the assay. Prior to performing each assay, the SYNCHRON system was calibrated, and a calibration curve was established. The normal reference range for concentrations of hsCRP using this high-sensitivity assay is 0.0–3.3 mg/L [33].

### 2.4. Statistical Analyses

Clinical characteristics of subjects were examined for differences between subjects with PAD and controls using independent *t*-tests for measurement variables and one degree of freedom Chi square tests for dichotomous variables. Preliminary examination of response variables revealed large deviations from normal distribution and, therefore, statistical methods not based on this assumption were used for hypothesis testing. For each variable, values were summarized within both PAD and control groups by presenting median and interquartile range, and two groups were compared using Spearman correlation between the dichotomous group variable and each response variable. Note that this is equivalent in *P* value to Wilcoxon test for two groups. Furthermore, since smoking may have an influence on oxidative stress, it was of interest to also examine whether the relatively large prevalence of current smokers in the PAD group had an influence on the group comparisons of study variables. Thus, partial Spearman correlation controlling for current smoking was performed to compare groups.

## 3. Results

The clinical characteristics of subjects with PAD and controls are displayed in Table 1. By definition, the PAD group had a lower ABI (*P* < 0.001) than the control group. Additionally, the PAD group had a higher prevalence of current smoking (*P* = 0.002), hypertension (*P* < 0.001), diabetes (*P* < 0.001), obesity (*P* = 0.008), abdominal obesity (*P* < 0.001), and metabolic syndrome (*P* < 0.001) than the controls. Furthermore, the PAD group had greater body weight (*P* = 0.40), higher BMI (*P* = 0.006), lower 6-minute walk distance (*P* < 0.001), lower daily ambulatory activity as measured by fewer daily strides taken (*P* < 0.001), and ambulation at a slower average cadence (*P* < 0.001).

The measurements from cultured endothelial cells treated with sera are shown in Table 2. The PAD group had greater endothelial apoptosis (*P* < 0.001) and greater endothelial ROS production (*P* < 0.001) than the control group. The two groups were not significantly different on NF-*κ*B activity. After adjusting for current smoking status, the significance status results for the endothelial cell data were unchanged. The circulating inflammation, antioxidant capacity, and vascular biomarkers of subjects with PAD and controls are displayed in Table 3. The PAD group had lower antioxidant capacity measured by HORAC activity (*P* < 0.001); higher inflammatory and vascular measures of hsCRP (*P* < 0.001), IL-1b (*P* = 0.036), IL-8 (*P* < 0.001), SAA (*P* < 0.001), VCAM-1 (*P* < 0.001), adiponectin (*P* < 0.001), apolipoprotein B (*P* = 0.013), and apolipoprotein CIII (*P* = 0.035); lower VEGF-A (*P* < 0.001) and HGF (*P* < 0.001) than the control group. The two groups were not significantly different on the remaining variables. After adjusting for current smoking status, IL-1b was no longer significantly different between groups (*P* = 0.200), whereas the results of all other variables show that the significance status remained unchanged from the unadjusted results.

## 4. Discussion

The primary novel findings were that endothelial cell apoptosis was 164% higher in the PAD group than in the control group, and endothelial cell ROS production was 62% higher. Furthermore, in the circulation, the PAD group had lower antioxidant capacity, higher levels of inflammation as measured by numerous biomarkers, and lower angiogenic biomarkers of VEGF-A and HGF.

### 4.1. Greater Endothelial Apoptosis and ROS Production in Subjects with PAD

Our major finding was that apoptosis and ROS production by detector endothelial cells were higher upon treatment with sera from subjects with PAD compared to that induced by sera from healthy controls. The higher endothelial apoptosis and prooxidative status of the PAD group support previous work which found that endothelial function, measured by brachial artery flow-mediated dilation, is impaired in subjects with PAD [21, 24]. The fact that the PAD group had higher endothelial cell apoptosis and ROS production suggests that they are at high risk for progression of atherosclerosis [21] and increased risk of coronary events [15, 21], which contributes to their elevated rates of cardiovascular mortality [4, 5]. We recently found that subjects with PAD were not different on endothelial apoptosis, ROS production, and inflammation compared to non-PAD subjects who had high atherosclerotic burden [31]. Thus, it is likely that the greater levels of endothelial apoptosis and ROS production in the PAD group in the current study are due to a combination of having both overt PAD and a cluster of concomitant cardiovascular risk factors. It remains unclear whether risk factor modification can favorably impact endothelial apoptosis and oxidative status in subjects with PAD.

### 4.2. Lower Antioxidant Capacity and Greater Inflammation in Subjects with PAD

A novel aspect to the current investigation was that the subjects with PAD had lower HORAC levels than healthy controls, indicating that their circulating antioxidant capacity is relatively low. To our knowledge, this is the first study describing impaired HORAC levels in subjects with PAD, and our finding suggests that they may benefit from greater dietary antioxidant intake and antioxidant supplementation. We recently found that those with PAD were not different on HORAC levels compared to non-PAD subjects with high atherosclerotic burden [31]. Collectively, our studies suggest that the combination of having both PAD and numerous cardiovascular risk factors is detrimental to circulating antioxidant capacity.

Another primary finding in the current study was that the subjects with PAD had greater inflammation, as measured by hsCRP, IL-8, SAA, and VCAM-1, compared to the healthy controls [31, 34–36]. The elevated inflammatory measures in subjects with PAD, coupled with their greater level of endothelial oxidative stress, suggest that they are prone to have accelerated myopathy from damaged mitochondrial electron transport chain function, thereby reducing energy production and increasing apoptosis and sarcopenia [37, 38]. Overall, the observation that inflammatory measures are elevated in subjects with PAD emphasizes the need to develop interventions that will reduce inflammation. We have recently shown that higher levels of community-based, daily ambulatory activity are associated with lower levels of inflammation in subjects with PAD and claudication [39], suggesting that an exercise intervention program may be efficacious to treat inflammation in this population. Furthermore, the efficacy of pharmacologic therapy such as statin medications, cilostazol, and other medications to favorably alter inflammation of subjects with PAD and claudication should be better established.

### 4.3. Lower Angiogenic Measures in Subjects with PAD

Another key finding to the current investigation was that VEGF-A and HGF are lower in subjects with PAD and claudication compared to healthy controls. This supports our recent study that subjects with PAD had lower VEGF-A than non-PAD subjects with high atherosclerotic burden [31]. Collectively, our two studies suggest that the lower VEGF-A in subjects with PAD is primarily from PAD and not from concomitant atherosclerotic risk factors. VEGF-A and HGF are angiogenic growth factors, and VEGF-A is positively associated with capillary number [40–43]. The lower levels of VEGF-A and HGF in the subjects with PAD and claudication suggest that they have lower levels of angiogenesis, which supports previous work demonstrating that lower VEGF-A is associated with a lower capillary/fiber ratio in subjects with PAD [44].

### 4.4. Limitations

There are limitations to this study. A self-selection bias may exist regarding study participation, as subjects who participated in this trial were volunteers. Therefore, they may represent those who were more interested in participation, who had better access to transportation to the research center, and who had relatively better health than subjects who did not volunteer. Furthermore, the results of this study are only applicable to PAD subjects who are limited by claudication and may not be generalized to subjects with less severe or more severe PAD. Women and African-Americans are well represented, and typical risk factors for PAD such as smoking, diabetes, hypertension, dyslipidemia, and obesity are highly prevalent. Thus, in subjects with PAD and claudication, the findings of the present study are generalizable to the large proportion of men and women with PAD. Another limitation is that the sample size of the control group is relatively small due to budgetary constraints associated with performing the numerous analyses of the blood samples. However, we believe that the consequences of the small number of healthy controls were minimal because large and significant group differences were found for many of the variables.

The relatively small sample size for the healthy controls yields less precision of variable estimates for this group and increases the chance of not detecting actual difference for some variables. Finally, there are limitations associated with the design of the study. The comparison of the measures between the PAD group and the control group utilizes a cross-sectional design, which does not allow causality to be established.

### 4.5. Conclusion and Clinical Significance

In conclusion, subjects with PAD have greater endothelial apoptosis and oxidative stress than control subjects with low burden of comorbid conditions and cardiovascular risk factors. Furthermore, subjects with PAD have lower systemic antioxidant capacity and angiogenic measures and higher circulating inflammatory parameters. The clinical significance is that optimal medical management of cardiovascular risk factors in subjects with PAD and claudication may be an efficacious approach to improve endothelial function, as well as systemic inflammation and vascular and metabolic biomarkers. Low antioxidant capacity in subjects with PAD suggests that antioxidant therapy may be efficacious to improve endothelial function and systemic inflammation.

## Figures and Tables

**Table 1:** Clinical characteristics of subjects with peripheral artery disease (PAD) and controls.

Variables	Control group	PAD group	*P* value
Age (years)	70 (9)	64 (10)	0.014
Sex (% men)	50	49	0.923
Race (% Caucasian)	69	45	0.069
Current smoking (% yes)	0	40	0.002
Hypertension (% yes)	25	89	<0.001
Dyslipidemia (% yes)	81	93	0.104
Diabetes (% yes)	0	48	<0.001
Obesity (% yes)	13	47	0.008
Abdominal obesity (% yes)	13	57	0.001
Metabolic syndrome (% yes)	0	84	<0.001
Weight (kg)	74.2 (12.5)	84.1 (18.7)	0.040
Body mass index (kg/m^2^)	25.5 (3.1)	29.6 (5.9)	0.006
Ankle/brachial index	1.12 (0.06)	0.72 (0.25)	<0.001
Six-minute walk distance (m)	524 (103)	337 (96)	<0.001
Six-minute walk rating of perceived exertion (score)	13.9 (2.1)	13.5 (2.4)	0.451
Total strides (strides/day)	5526 (2381)	3306 (1611)	<0.001
Average cadence (strides/min)	15.9 (3.8)	11.7 (2.6)	<0.001

Values are means (standard deviation) or percentage of subjects in each group.

**Table 2:** Measurements from cultured endothelial cells treated with sera from subjects with peripheral artery disease (PAD) and controls.

Variables	Control group	PAD group	*P* value
Apoptosis (arbitrary units)	0.42 (0.43)	1.11 (0.35)	<0.001
Cellular ROS production (arbitrary units)	17.00 (2.92)	27.55 (8.82)	<0.001
NF-*κ*B activity (arbitrary units)	1.12 (0.55)	1.20 (0.87)	0.866

ROS: reactive oxygen species.

Values are medians (interquartile ranges).

**Table 3:** Circulating inflammatory, antioxidant capacity, and vascular biomarkers of subjects with peripheral artery disease (PAD) and controls.

Variables	Control group	PAD group	*P* value
C-reactive protein (mg/L)	0.80 (1.20)	4.10 (5.96)	<0.001
Tissue necrosis factor alpha (pg/mL)	46 (18)	52 (26)	0.414
Interleukin-1b (pg/mL)	19 (7)	16 (5)	0.036
Interleukin-6 (pg/mL)	21 (12)	24 (12)	0.539
Interleukin-8 (pg/mL)	58 (26)	96 (56)	<0.001
Monocyte chemotactic protein-1 (pg/mL)	1102 (1135)	1146 (1712)	0.489
Myeloperoxidase (pg/mL)	525 (1285)	28 (39)	0.145
Matrix metallopeptidase-9 (pg/mL)	1035 (1189)	685 (311)	0.398
Serum amyloid A (pg/mL)	5563 (2253)	9335 (3818)	<0.001
Hydroxyl Radical Antioxidant Capacity (arbitrary units)	1.91 (0.77)	0.95 (0.24)	<0.001
E selectin (pg/mL)	49 (63)	40 (49)	0.412
Vascular cell adhesion molecule-1 (pg/mL)	1010 (568)	2164 (876)	<0.001
Intercellular cell adhesion molecule-1 (pg/mL)	1874 (2704)	1936 (1294)	0.763
Vascular endothelial growth factor-A (pg/mL)	52 (114)	29 (34)	<0.001
Leptin (pg/mL)	721 (3068)	1974 (3032)	0.214
Adiponectin (pg/mL)	3450 (653)	5540 (1501)	<0.001
Plasminogen activator inhibitor-1 (ng/mL)	497 (1084)	726 (468)	0.595
Apolipoprotein B (ng/mL)	23 (63)	66 (57)	0.013
Apolipoprotein CIII (ng/mL)	928 (822)	1336 (906)	0.035
Hepatocyte growth factor (pg/mL)	126 (103)	71 (38)	<0.001
Nerve growth factor (pg/mL)	12 (3)	14 (8)	0.060

Values are medians (interquartile ranges).
